# A lightweight deep convolutional neural network development for soybean leaf disease recognition

**DOI:** 10.3389/fpls.2025.1655564

**Published:** 2025-09-30

**Authors:** Yakun Zhang, Ruofei Bao, Mengxin Guan, Zixuan Wang, Libo Wang, Xiahua Cui, Xiaoli Niu, Yan Wang, Shaukat Ali, Yafei Wang

**Affiliations:** ^1^ Department College of Agricultural Equipment Engineering, Organization Henan University of Science and Technology, Luoyang, China; ^2^ Department College of Food Bioengineering, Organization Henan University of Science and Technology, Luoyang, China; ^3^ Department College of Biological and Agricultural Engineering, Organization Jilin University, Changchun, China; ^4^ Department Wah Engineering College, Organization University of Wah, Wah Cantt, Pakistan; ^5^ Department School of Agricultural Engineering, Organization Jiangsu University, Zhenjiang, China

**Keywords:** soybean leaf diseases, disease diagnosis, multiscale feature extraction fusion, dense connectivity, deep convolutional neural networks

## Abstract

Soybean is one of the world’s major oil-bearing crops and occupies an important role in the daily diet of human beings. However, the frequent occurrence of soybean leaf diseases caused serious threats to its yield and quality during soybean cultivation. Rapid identification of soybean leaf diseases could provide a better solution for efficient control and subsequent precision application. In this study, a lightweight deep convolutional neural network (CNN) based on multiscale feature extraction fusion (MFEF) and combined with a dense connectivity (DC) network (MFEF-DCNet) was proposed for soybean leaf disease identification. In MFEF-DCNet, a multiscale feature extraction fusion (MFEF) module for soybean leaves was constructed by utilizing a convolutional attention module and depth-separable convolution to improve the model feature extraction capability. Multiscale features are fused by using dense connections (DC) in the backbone network to improve the model generalization capability. Experiments were implemented on eight distinct disease and deficiency classes of soybean images (including bacterial blight, cercospora leaf blight, downy mildew, frogeye leaf spot, healthy, potassium deficiency, soybean rust, and target spot) using the proposed network. The results showed that the MFEF-DCNet had an accuracy of 0.9470, an average precision of 0.9510, an average recall of 0.9480, and an F1-score of 0.9490 for soybean leaf disease identification. And MFEF-DCNet had certain performance advantages in terms of classification accuracy, convergence speed and other effects compared with VGG16, ResNet50, DenseNet201, EfficientNetB0, Xception and MobileNetV3_small models. In addition, the accuracy of the MFEF-DCNet model in recognizing soybean diseases in local data was 0.9024, which indicated that the MFEF-DCNet model had favorable application in practical applications. The proposed model and experience in this study could provide useful inspiration for automated disease identification in soybean and other crops.

## 1 Introduction

Soybeans are one of the important crops with high protein, high oil, and high nutrition characteristics, widely used in food, feed, industry and other fields (Sun et al., 2018) (Zhu et al., 2019), (Huang et al., 2019). Soybeans are usually processed into protein rich foods or edible oils, which is rich in proteins. In addition, soybean protein isolate hydrogel extracted from soybean contains nearly 20 kinds of essential amino acids for human body (Wang et al., 2023; Ding et al., 2021; Han et al., 2016). Soybean oil can be used as bio-petroleum after chemical conversion, which generates more energy than fossil energy sources (Pradhan et al., 2010). However, in the process of soybean cultivation, the frequent occurrence of soybean leaf diseases has caused huge economic losses, as well as posing a great threat to human health and food security. Soybean yield losses due to disease account for about 8-25% of total soybean production average annual (Savary et al., 2019). In addition to these direct losses, problems caused by the extensive use of pesticides for diseases, such as environmental pollution, soil fertility degradation and increased resistance, have become important obstacles to the sustainable cultivation of soybeans. During the soybean growing process, the types of soybean diseases could be accurately recognized in time and agricultural chemicals could be applied accordingly. Then soybean cultivation will develop in a sustainable direction to better meet the world’s demand for soybeans. The kinds of soybean diseases can be manifested on the leaves, such as discoloration, spots, holes, etc. These diseases can be identified by experienced farmers or experts based on disease characteristics. However, in general, it is difficult to achieve rapid and accurate diagnosis of soybean diseases due to the lack of relevant professional experience and disease identification knowledge among soybean farmers, which has become a bottleneck restricting the development of the soybean industry.

With the advancements in computer vision and deep learning techniques, automated recognition of images has made significant progress in terms of accuracy, recognition efficiency and economic utility. In recent years, various methods had been attempted to be applied to automated crop disease diagnosis, including K-nearest neighbor classifier (Chen et al., 2025), color transformation and histogram methods (Rachmad et al., 2023), support vector machine (SVM) (Chen et al., 2024; Sun et al., 2016; Wang et al., 2022), convolutional neural network (CNN) (Mahmood ur Rehman et al., 2024; Qiu et al., 2024; Sun et al., 2018). In terms of disease recognition performance, CNN was currently the most effective.

CNN (Zhang and Dai, 2025) is a special class of artificial neural networks with the main characteristics of convolutional operation compared with other neural network models (Zhiming et al. 2024; Weidong et al. 2022). CNN combines the advantages of convolutional kernel local feature extraction and BP neural network back propagation. Through local feature extraction and backward weight sharing operation, CNN achieves the reduction of the number of parameters and the improvement of network generalization ability (Shengyi et al. 2021). Currently, convolutional neural networks have great potential in agriculture, especially in agricultural image processing (Archana and Jeevaraj, 2024; El Sakka et al., 2025; Jiehong et al. 2023). In response to the difficulty of effectively extracting soybean diseased leaves from complex backgrounds, Aditya et al. proposed a SoyNet model based on computer vision and convolutional neural network, which realized the effective separation of soybean diseased leaves from complex backgrounds, with a model classification accuracy of 98.14% (Karlekar and Seal, 2020). Wu et al. developed an enhanced deep learning network model for predictive classification of soybean leaf diseases with an average recognition accuracy of 85.42% through feature extraction, attention computation and classification (Wu et al., 2023). Sandeep et al. developed a novel deep learning model based on convolutional neural networks for classification of soybean leaf pests and diseases, which is also capable of disease level prediction (Goshika et al., 2023). Vivek et al. designed the SoyaTrans model by combining the CNN architecture with a swin-transformer, which improves the classification performance and reduces the complexity of the model by introducing a new random shift mechanism (Sharma et al., 2025).

However, most of the existing soybean leaf disease identification methods suffer from complex models, time-consuming, and computational resource-consuming problems, which made these methods difficult to satisfy the production demands for rapid disease detection, especially when deployed and implemented on mobile devices. Therefore, the focus of this study was to improve the convergence speed and model lightweight of convolutional neural networks while ensuring the recognition accuracy. The goal of this study was to develop a lightweight automatic classifier for digital images of soybean diseases based on CNN. By using multi-scale feature extraction and fusion modules, the classifier can better extract feature information from images. And data expansion method was used to balance the data samples between each category to prevent model overfitting. An image enhancement algorithm was also used to enhance the input soybean disease images to improve the applicability of the classifier. The soybean disease identification network designed in the study can accurately identify common soybean leaf disease categories for efficient and accurate classification and decision making.

## 2 Materials and methods

### 2.1 Database set

**Figure 1:**
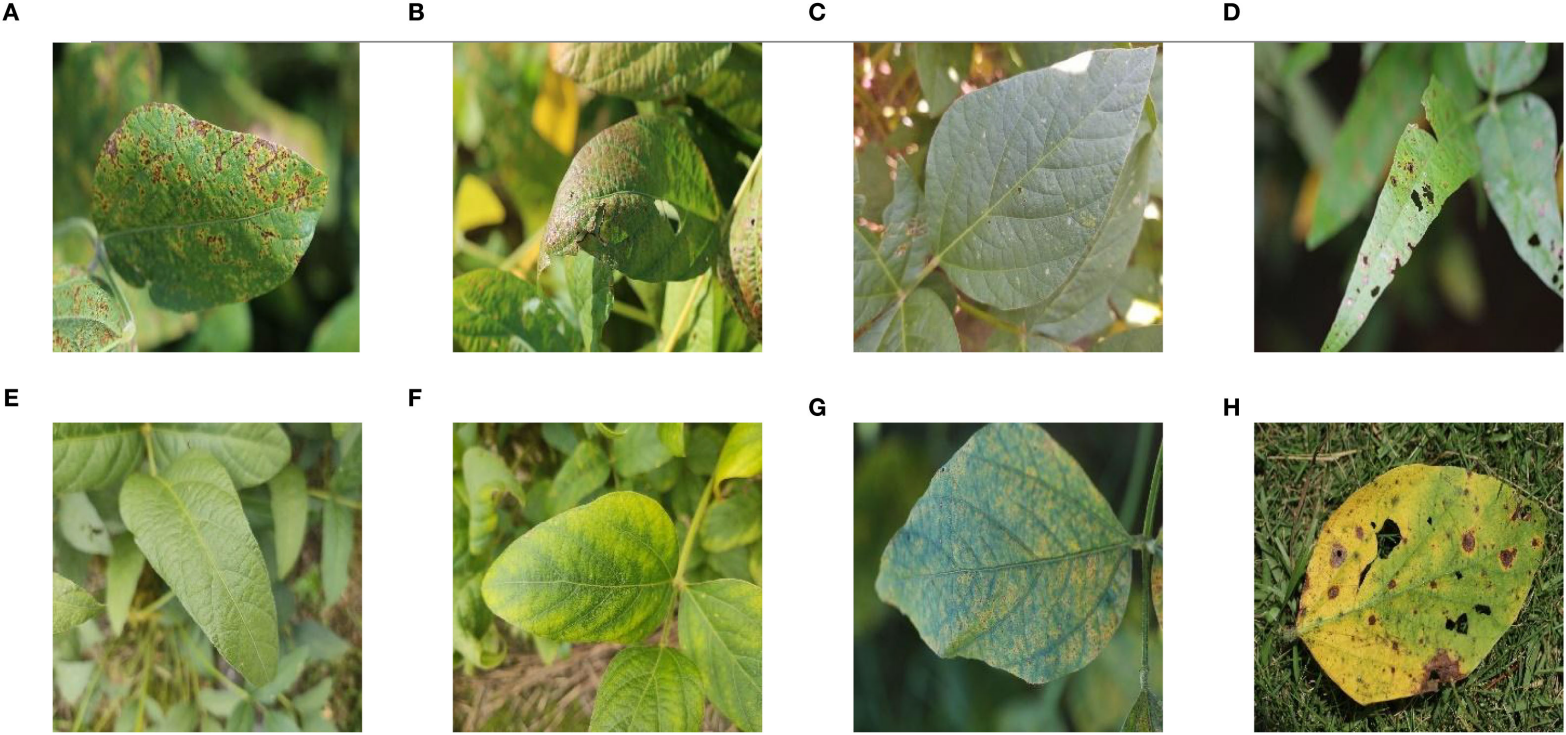
Example images of eight soybean leaf diseases: **(A)** Example image of soybean leaf with bacterial blight; **(B)** Example image of soybean leaf with cercospora leaf blight; **(C)** Example image of soybean leaf with downey mildew; **(D)** Example image of soybean leaf with frogeye leaf spot; **(E)** Example image of soybean healthy leaf; **(F)** Example image of soybean leaf with potassium deficiency; **(G)** Example image of soybean leaf with rust; **(H)** Example image of soybean leaf with target spot.

The dataset used for the study was the Auburn Soybean Disease Image Dataset (ASDID), which is publicly available and has been extensively studied and validated (Bevers et al., 2022). The dataset contains 9648 soybean leaf images, of which 4,181 were taken in 2020 and 5,467 in 2021. The dataset contains eight main categories: bacterial blight (**Figure 1A**), cercospora leaf blight (**Figure 1B**), downey mildew (**Figure 1C**), frogeye leaf spot (**Figure 1D**), healthy (**Figure 1E**), potassium deficiency (**Figure 1F**), soybean rust (**Figure 1G**), target spot (**Figure 1H**). The ASDID dataset were used to construct soybean disease identification model.

The original 9648 images were randomly divided in the ratio of 9:1, of which 8686 were used for model training and validation, and 962 were used for model testing. **Table 1** showed the division of the data according to the 9:1 ratio. **Figure 1** showed examples of soybean leaves photographed for all eight disease categories.

**Table 1:** Division of original data.

Category	Number of original images	Number of training and validation images	Number of test images
Bacterial blight	484	436	48
Cercospora leaf blight	1598	1439	159
Downey mildew	652	587	65
Frogeye leaf spot	1540	1386	154
Healthy	1632	1469	163
Potassium deficiency	1034	931	103
Soybean rust	1627	1465	162
Target spot	1081	973	108
Total	9648	8686	962

In addition, in order to verify the adaptability and local application effect of the constructed model, 164 images of four categories of soybean leaves were collected at Dayuzhuang, Lanqing Township, Zhengyang County, Zhumadian City, Henan Province (32027’ 37’’N, 11420’ 11’’E). There were 48 images of bacterial blight, 52 images of frogeye leaf spot disease, 35 images of healthy leaves and 29 images of soybean rust. Soybean leaf images were collected on August 22, 2020. After completing the construction and training of the MFEF-DCNet model, the collected images of soybean leaves were input into the trained MFEF-DCNet model to test the application effect of the model in practice.

### 2.2 Data balance

As shown in **Table 1**, the number of healthy samples used for training and validation was 1469, while the number of bacterial blight was 436. The ratio of the number of healthy samples to the number of bacterial blight samples was greater than 3:1, indicating that the distribution of samples shown a serious imbalance. In CNN, imbalance in sample distribution may lead to overfitting problems in network models. In addition, sample data with differences between different categories could severely affect the accuracy of network training and limit network performance (Buda et al., 2018). Therefore, a data balancing algorithm was introduced before using the data for training (Gao et al., 2021).

The above sampling method was used to balance the sample data, which enhanced the generalization ability of the network. The method was as follows:

(1) Index building: Firstly, the expression of the datasets was defined as $D=\{[M_{0},M_{l},M_{2},\cdots M_{i}]{\left[N_{0},N_{l},N_{2},\cdots N_{i}\right]}^{T}\}$ , in which $M_{i}$ represented the type of soybean leaf disease, and $N_{i}$ was the number of samples which corresponded to $M_{i}$ . Then the maximum number ${\text{N}}_{\text{i}}$ of samples in the datasets and its corresponding $M_{i}$ were counted. Finally, the maximum value $N_{i}$ was expressed by $N_{max}$ , while $M_{i}\{i\in [0,7]\}$ marked the type which corresponded to $N_{max}$ , in which i expressed the label serial number (Label 0 indicated bacterial blight, Label 1 indicated cercospora leaf blight, Label 2 indicated downey mildew, Label 3 indicated frogeye leaf spot, Label 4 indicated healthy, Label 5 indicated potassium deficient, Label 6 indicated soybean rust, Label 7 indicated target spot).

(2) Calculation of the proportionality constant C: $N_{max}$ was chosen as the numerator and $N_{i}$ was selected as the denominator. The constant C was calculated according to Equation 1.


$${\text{C}}_{\text{i}}=\frac{{\text{N}}_{\text{max}}}{{\text{N}}_{\text{i}}}(\text{i}\in [0,7],\text{Floor})$$

(3) Up-sampling: the proportionality factor $C=\{C_{0},C_{1}\cdots C_{i}\}$ was obtained from Equation 1. Smaller numbers of samples were up-sampled using different methods based on the proportionality constant. Data balancing was achieved by complex up-sampling, which is implemented by taking different up-sampling measures according to the size of the proportionality factor $C_{i}$ , as shown in Equation 2:


$$\begin{array}{c} \{\frac{0≦/{\text{C}}_{\text{i}}≦/1,\text{Dataset}\;\text{uchanged}}{1<{\text{C}}_{\text{i}}<3,\text{Rotation}}\\ \{\frac{3≦/{\text{C}}_{\text{i}}<4,\text{Rotation},\;\text{Contrast}}{4≦/{\text{C}}_{\text{i}}<5,\text{Sharpness},\;\text{Rotation},\;\text{Contrast}} \end{array}$$

(4) Exportation: $D^{\prime }=\{[M_{0},M_{1},M_{2},\cdot \cdot \cdot \cdot M_{i}]{\left[N_{0}^{'},N_{1}^{'},N_{2}^{'},\cdot \cdot \cdot \cdot N_{i}^{'},\right]}^{T}\}$ was the balanced output, where $\;N_{i}^{'}$ was calculated from Equation 3.


$${\text{N}}_{\text{i}}^{'}={\text{N}}_{\text{i}}\times {\text{C}}_{\text{i}}(\text{i}\in [0,7])$$

In the calculated datasets, D={[*M_0_* indicated bacterial spot, *M_1_* indicated caecilian leaf blight, *M_2_* indicated downy mildew, *M_3_* indicated frogeye leaf spot, *M_4_* indicated healthy, *M_5_* indicated potassium deficiency, *M_6_* indicated rust, *M_7_* indicated target spot][*N_0_* = 436, *N_l_* = 1439, *N_2_* = 587, *N_3_* = 1386, *N_4_* = 1469, *N_5_* = 931, *N_6_* = 1465, *N_7_* = 973]^T^}, the sample number of bacterial blight was the lowest while healthy sample number leaf sample was the highest.

Since $N_{\text{max}}$ was 1469, $C_{0}$ was equal to 3 according to Equation 1, 2. That means 3 was calculated by dividing 1469 by 436 using the floor principle. The bacterial blight dataset was balanced by an increase of 200% in the number of bacterial blight samples through rotation and contrast adjustments.

According to the above method, the proportionality constants for each disease type sample were calculated based on the data in **Table 2**. On the basis of Equation 3, $N_{0}^{"}$ , $N_{1}^{"}$ , $N_{2}^{"}$ , $N_{3}^{"}$ , $N_{4}^{"}$ , $N_{5}^{"}$ , $N_{6}^{"}$ , $N_{7}^{"}$ were obtained as follows:

**Table 2:** Number of categories before and after data balance.

Category	Number of original images	Constant Ci	Balance the quantity	Total number after balance
Bacterial blight	436	3	872	1380
Cercospora leaf blight	1439	1	0	1439
Downey mildew	587	2	587	1174
Frogeye leaf spot	1386	1	0	1386
Healthy	1469	1	0	1469
Potassium deficiency	931	1	0	931
Soybean rust	1465	1	0	1465
Target spot	973	1	0	973
Total	8686			10145


$${\text{N}}_{0}^{'}={\text{N}}_{0}\cdot {\text{C}}_{0}=436\times 3=1308$$

$${\text{N}}_{1}^{'}={\text{N}}_{1}\cdot {\text{C}}_{1}=1439\times 1=1439$$

$${\text{N}}_{2}^{'}={\text{N}}_{2}\cdot {\text{C}}_{2}=587\times 2=1174$$

$${\text{N}}_{3}^{'}={\text{N}}_{3}\cdot {\text{C}}_{3}=1386\times 1=1386$$

$${\text{N}}_{4}^{'}={\text{N}}_{4}\cdot {\text{C}}_{4}=1469\times 1=1469$$

$${\text{N}}_{5}^{'}={\text{N}}_{5}\cdot {\text{C}}_{5}=931\times 1=931\;$$

$${\text{N}}_{6}^{'}={\text{N}}_{6}\cdot {\text{C}}_{6}=1465\times 1=1465$$

$${\text{N}}_{7}^{'}={\text{N}}_{7}\cdot {\text{C}}_{7}=973\times 1=973$$

**Table 2** displayed the sample distribution of each dataset type after data balancing. As shown in **Table 2**, the maximum number of healthy leaves after data balancing was 1469 and the minimum number of potassium deficient leaves after data balancing was 931. The ratio of the two sample datasets was approximately 1:1.57, which indicated a more balanced distribution of sample data.

When the number of images in each category was roughly the equal, the model could better learn the features of each category and avoid overfitting or underfitting of some categories.

### 2.3 Data enhancement

**Figure 2:**
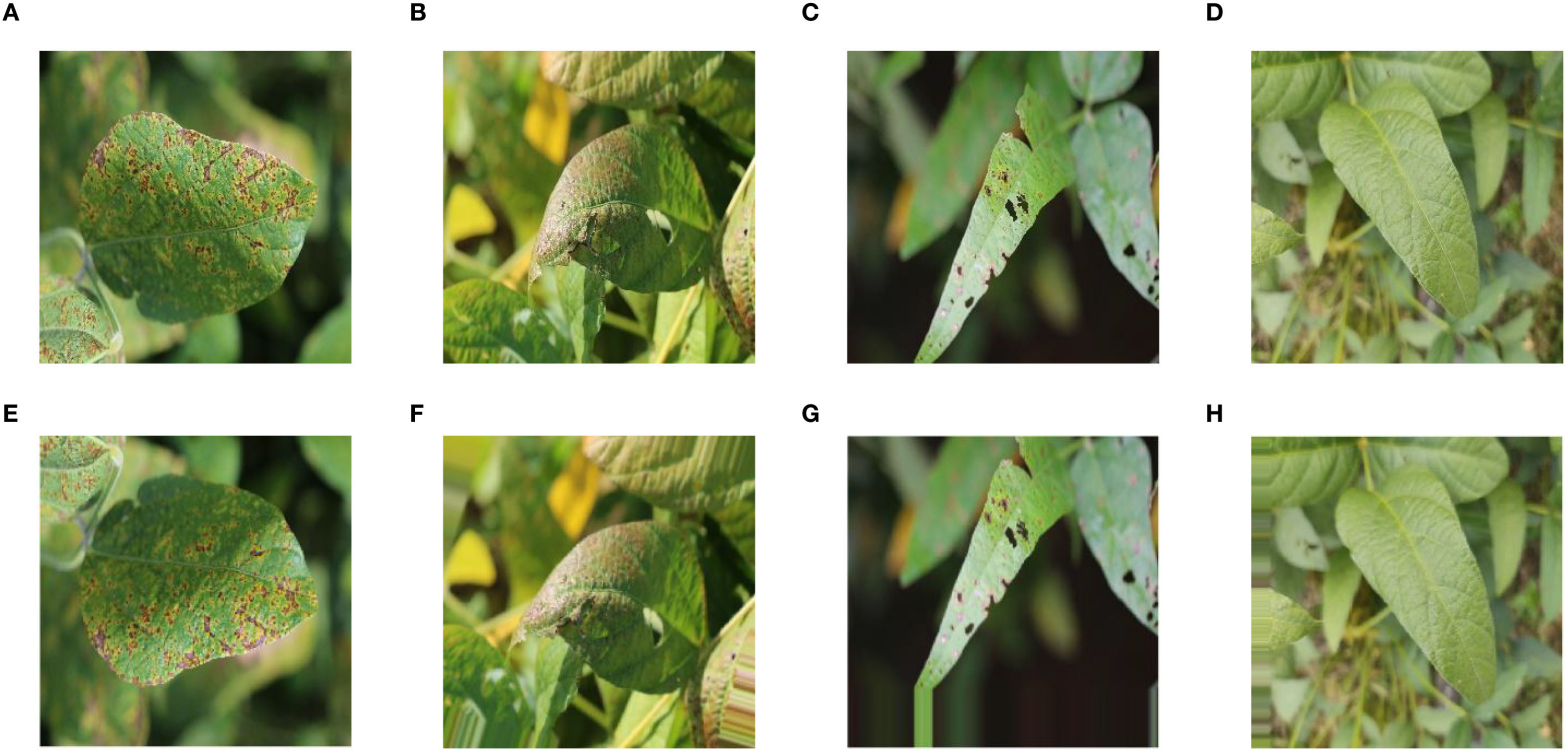
Comparison before and after image enhancement: **(A)** Origional image of soybean leaf with bacterial blight; **(B)** Origional image of soybean leaf with cercospora leaf blight; **(C)** Origional image of soybean leaf with downey mildew; **(D)** Origional image of soybean healthy leaf; **(E)** Image obtained after flip for original image of soybean leaf with bacterial blight; **(F)** Image obtained after rotation for original image of soybean leaf with cercospora leaf blight; **(G)** Image obtained after moving vertically for original image of soybean leaf with downey mildew; **(H)** Image obtained after moving horizontally for original image of soybean healthy leaf.

Image enhancement was a conventional data preprocessing method used to improve inter-class parity and increase the size of training samples. This method was accomplished by employing various transformations such as rotating, scaling, reflecting, adjusting brightness, and adding noise to the original image. ImageDataGenerator in Keras was able to enhance the data in a fast way (Abadi et al., 2016). Therefore, in the study, ImageDataGenerator was used to perform shift, brightness change, flip, rotate, and normalization operations on the data in the training set and normalization operations on the data in the validation set. **Figure 2** showed the effect comparison before and after image enhancement of soybean leaves. **Figure 2A** showed the original image of soybean leaf with bacterial blight; **Figure 2B** showed the original image of soybean leaf with cercospora leaf blight; **Figure 2C** showed the original image of soybean leaf with downey mildew; **Figure 2D** showed the original image of soybean healthy leaf; **Figure 2E** showed the image obtained after flip for original image of soybean leaf with bacterial blight; **Figure 2F** showed the image obtained after rotation for original image of soybean leaf with cercospora leaf blight; **Figure 2G** showed the image obtained after moving vertically for original image of soybean leaf with downey mildew; **Figure 2H** showed the image obtained after moving horizontally for original image of soybean healthy leaf.

### 2.4 Model design

#### 2.4.1 Block construction

The attention mechanism was a technology inspired by the human visual and cognitive system, which allowed CNN to focus on processing the relevant parts of the input data. By using the attention mechanism, CNN could automatically learn and focus on the important information in the input, thus improving the generalization ability of the model. Convolutional Block Attention Module (CBAM) was a simple and effective lightweight attention module for feed-forward CNN, which consisted of a channel attention module (CAM) and a spatial attention module (SAM) (Woo et al., 2018).

CAM was used to learn the channel weights using a shared fully connected layer and activation functions. SAM was used to compute the spatial weights of the feature maps using the maximum pool and the average pool. Thus, the CBAM module was able extract the feature information in the feature map more efficiently when training the neural network. CBAM could be used in various CNN architectures, and has been widely applied with remarkable performance to tasks such as image classification (Guo et al., 2025), target detection (Pei, 2022), instance segmentation (Ma et al., 2024).

**Figure 3:**
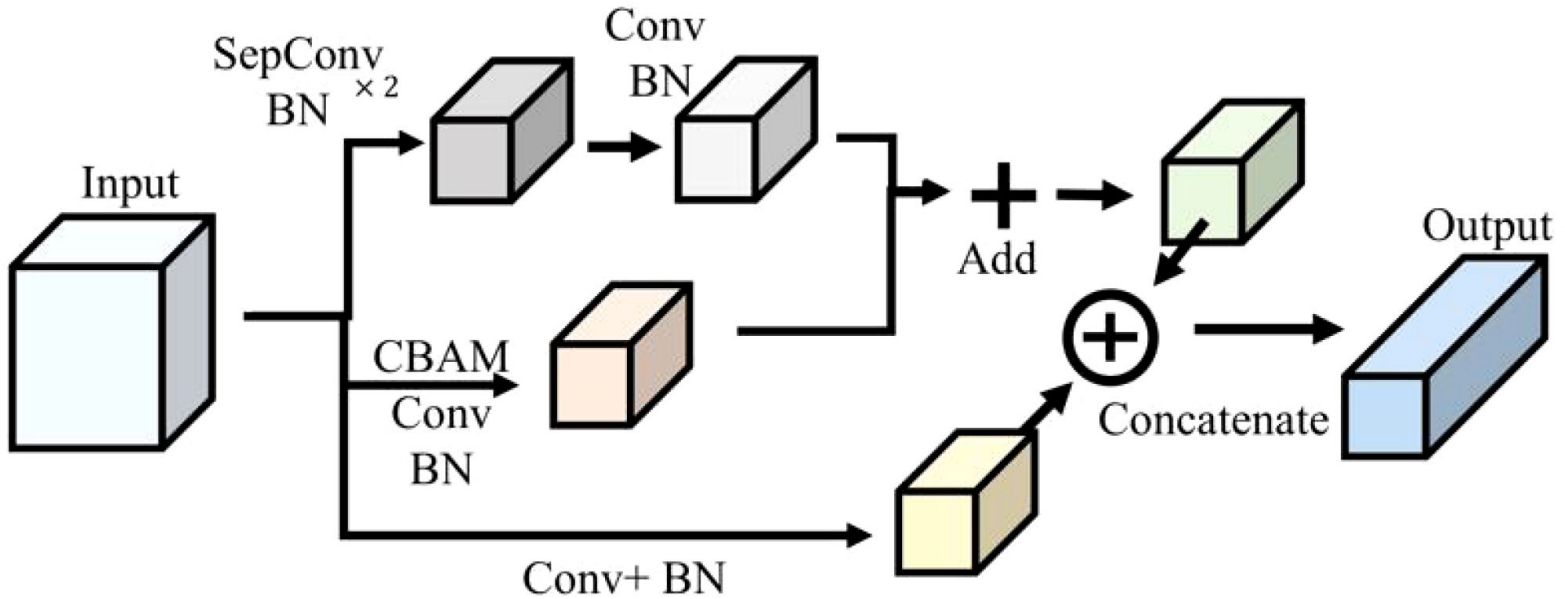
Multiscale feature extraction and fusion for soybean leaves.

The multiscale feature extraction and fusion for soybean leaves (MFEF-SL) module was constructed in this research. On the one hand, the input feature layer was extracted by two depthwise separable convolutions and batch normalization, and then the feature layer was reduced by maximum pooling. On the other hand, the feature filtering and size adjustment of the feature map were realized by convolution attention module, ordinary convolution and batch normalization. In the network structure, the module original input feature layer was spliced in the channel direction after maximum pooling and batch normalization. The merging of the above two aspects was realized through the summation of the corresponding elements, and the output was finally obtained. The specific network structure connection was shown in **Figure 3**.

#### 2.4.2 MFEF-DCNet

DenseNet was a densely connected neural network architecture (Huang et al., 2017). This network architecture was realized by establishing direct connections between the outputs of each layer and the inputs of all subsequent layers, whereby the outputs of each layer were directly connected to all previous layers. The dense connection allowed the output of each layer to be used directly by the subsequent layers. This approach improved the efficiency of feature transfer, which in turn enhanced the convergence speed and accuracy of the model (Zhang et al., 2024). DenseNet has achieved outstanding performance in various image classification and target detection tasks (Zhang et al., 2025). Meanwhile, the idea of dense connection was also used in the architecture of model design (Xu et al., 2024). It exhibited strong feature extraction and usage capabilities while maintaining computational efficiency.

**Figure 4:**
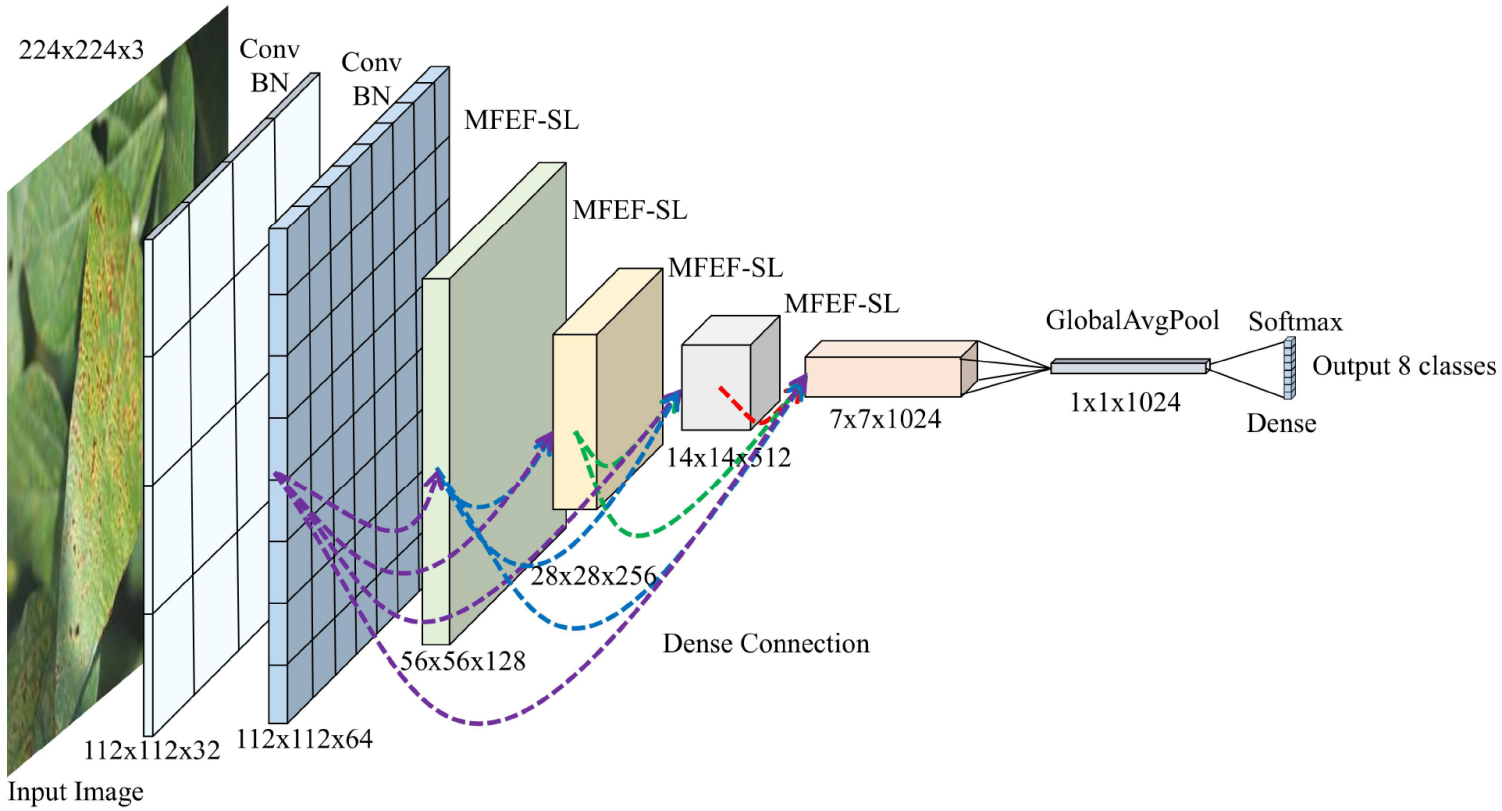
MFEF-DCNet.

The multiscale feature extraction fusion dense connected network (MFEF-DCNet) for soybean leaves proposed in this study was mainly constructed by the modules of convolutional layer, MFEF-SL, global average pooling and fully connected layer. The network structure was shown in **Figure 4**.

Firstly, the input image was subjected to initial feature extraction by two convolutions. Secondly, higher latitude feature extraction was performed by four MFEF-SL module operations. For high-latitude feature extraction, dense connection was used between MFEF-SL Blocks. Namely, the input of each block was the splicing result after pooling the output of the previous block. In addition, due to the fact that the size of the feature map was halved after the operation of the MFEF-SL modules, the same image size was ensured by a maximum pooling operation before performing a dense join, as shown by the dotted line join in **Figure 4**. Thirdly, a global average pooling and a fully connected layer of eight neurons were performed. Finally, the probabilities of the eight categories are output after passing the Softmax activation function.

#### 2.4.3 Training

During model training, a stochastic gradient descent optimizer was used to reduce the loss values. The learning rate set was 1e-4, momentum was 0.9, loss function was categorical cross entropy, batch size was 32, and the initial training epoch was 100. All the models were trained from the scratch.

#### 2.4.4 Evaluation

The performance of a model was usually evaluated using test set data. Therefore 962 images from the original dataset were used to exam the performance of the model. Model evaluation indexes, such as accuracy, precision, Recall, F1-Score, and the corresponding macro average and weighted average, were used for model evaluation. Accuracy is usually considered as the overall performance of the model and is used to calculate the correctly recognized labels.


$$\text{Accuracy}=\frac{\text{TP}+\text{TN}}{\text{TP}+\text{TN}+\text{FP}+\text{FN}}$$

Precision (P) is used to assess the accuracy of the recognitions by comparing the number of correctly recognized images for a disease category to the total number of images recognized by the model for that category.


$$\text{Precision}=\frac{\text{TP}}{\text{TP}+\text{FP}}$$

Recall (R) is used to measure the ability of the model to cover positive class samples, and is defined as the number of correctly recognized positive samples divided by the total number of positive samples.


$$\text{Recall}=\frac{\text{TP}}{\text{TP}+\text{FN}}$$

F1-Score (F) is the weighted harmonic mean of precision and recall. When the F1-Score is closer to 100% it indicates that the recognition performance of the model is better.


$$\text{F}1-\text{Score}=\frac{2\times \text{Precision}\times \text{Recall}}{\text{Precision}+\text{Recall}}\times 100\%$$

In the above equation, TP represents True Positive, FP represents False Positive, TN represents True Negative, and FN represents False Negative.

Macro average is calculated by averaging the precision, recall and F1 score for each category. Thus, macro precision, macro recall and macro F1 score are calculated. However, when there is a serious category imbalance in the dataset, it is not appropriate to use macro average as an evaluation index, instead weighted average is used as the evaluation index. Weighted average assigns different weights to each class based on the ratio of the sample size of each class to the total sample size. The corresponding weighted precision, weighted recall, and weighted F1-Score are then calculated based on the weights.

## 3 Results

### 3.1 Model performance comparison

**Table 3** displayed the training results and model parameter information based on the MFEF-DCNet, MobileNetV3_small, EfficientNetB0, VGG16, DenseNet201, Xception, and ResNet50 models. The training of all these models was trained from the scratch. In the table, the total parameters were the sum of trainable and non-trainable parameters, which was a reference indicator of the model capacity. The non-trainable parameters were usually set when the model was built and remained constant throughout the training process. During the training process, the trainable parameters were adjusted according to the training data to enable the model to fit the data better. It could be seen from the table that MobileNetV3_small had the lowest single epoch training time among all models. However, the final performance of MobileNetV3_small was poor with an accuracy of 0.9013. Therefore, MobileNetV3_small was not able to meet the practical requirements. Although MFEF-DCNet was longer than MobileNetV3_small in terms of training time for a single epoch, the model achieves a test precision of 0.947 due to the well-designed and optimized structure of MFEF-DCNet. In addition, although the MFEF-DCNet model was the most lightweight with a minimum total parameter of 1,200,104, the values of precision, recall and F1 score in MFEF-DCNet were exceed 0.95. Therefore, the comprehensive performance of MFEF-DCNet was the best among the all mentioned models.

**Table 3:** Number of categories before and after data balance.

Model Name.	Total Params	Trainable Params	Non-trainable params	Accuracy	Precision	Recall	F1	Average Training Latency (secs/epoch)
MFEF-DCNet	1,200,104	1,188,776	11328	0.9551	0.9608	0.9572	0.9581	156.88
EfficientNetB0	4,222,315	4,180,292	42,023	0.9453	0.9505	0.9452	0.9463	160.97
ResNet50	23,858,760	23,805,640	53,120	0.9307	0.9322	0.9349	0.9331	165.34
VGG16	14,789,128	14,789,128	0	0.9253	0.9261	0.9304	0.9268	162.67
Xception	21,132,528	21,078,000	54,528	0.9298	0.9416	0.9341	0.9351	172.83
MobileNetV3_small	1,526,056	1,513,944	12,112	0.9013	0.9096	0.9037	0.9054	32.16
DenseNet201	18,576,648	18,347,592	229,056	0.8738	0.9016	0.8761	0.8819	183.74

**Figure 5:**
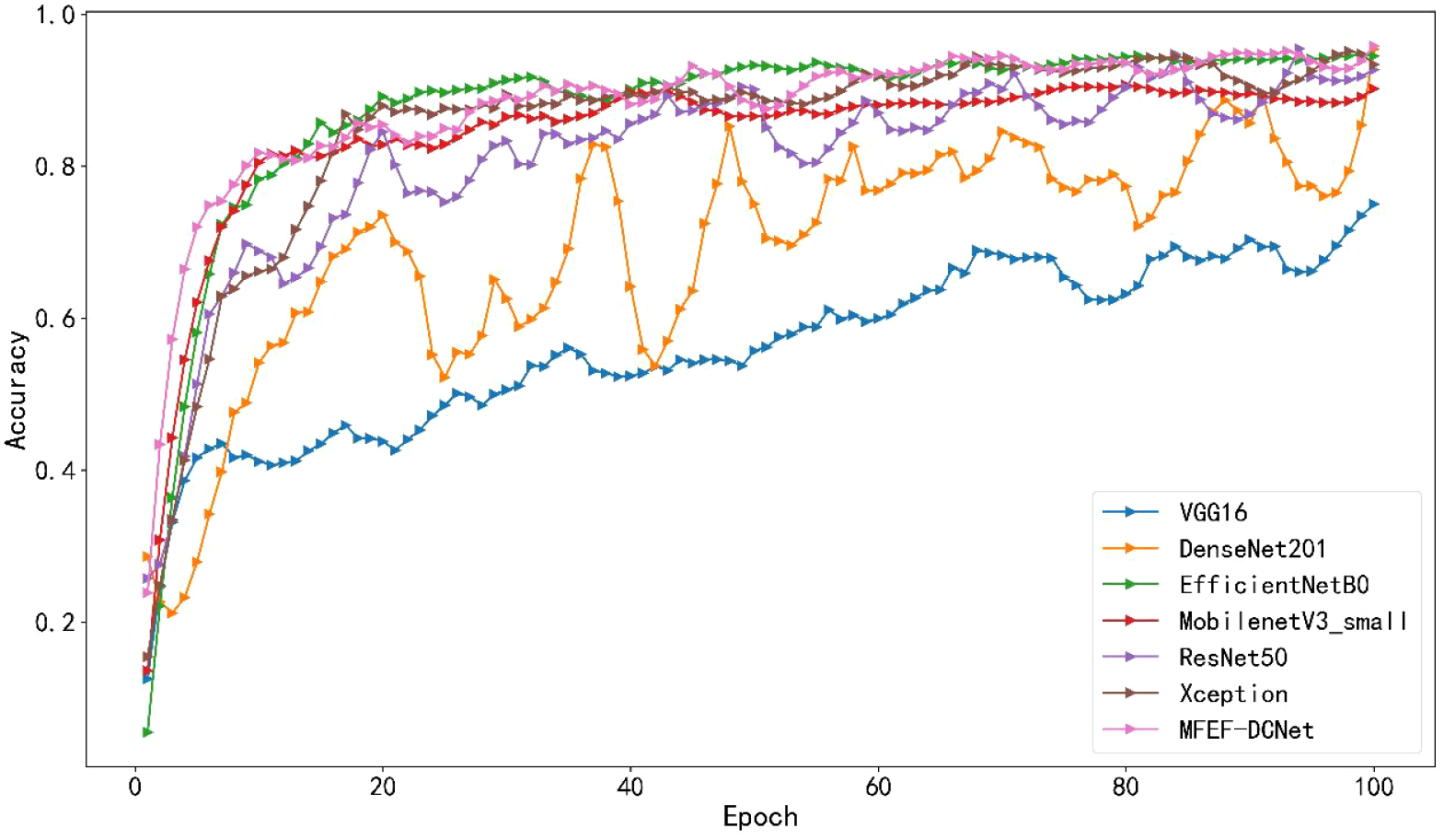
Accuracy curves of different models.

**Figure 6:**
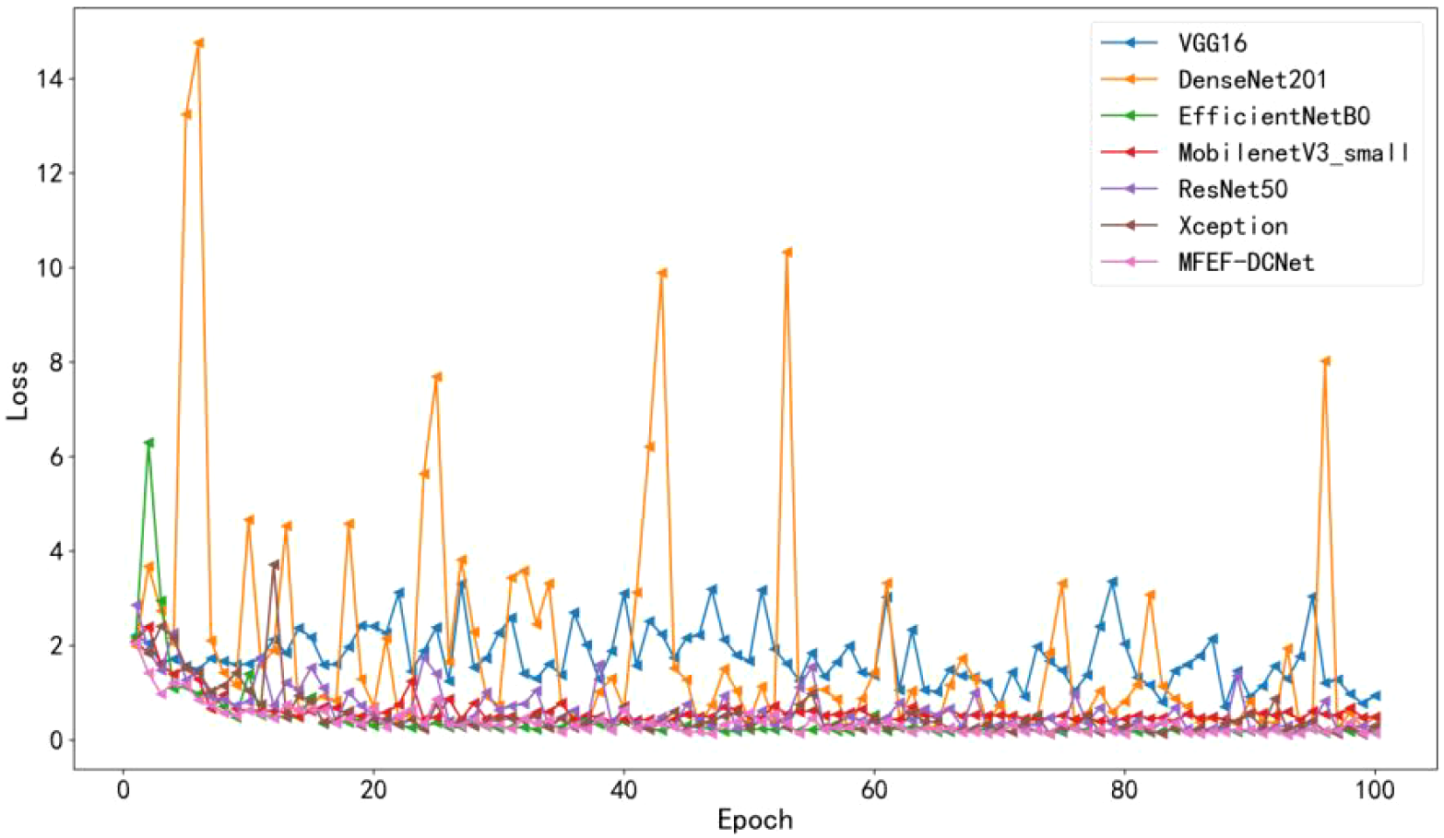
Loss value curves of different models.

The accuracy curves comparison for the training process of all models were depicted in **Figure 5**. By analyzing the accuracy curve of MobileNetV3_small, it can be found that the training accuracy was increasing at the beginning. After 30 epochs, the training accuracy tended to stabilize. However, the final accuracy of MobileNetV3_small was low, which cannot satisfy the requirements of real production environment. In addition, it can be observed that MFEF-DCNet shown the fastest convergence rate in the first 10 epochs from **Figure 5**. Meanwhile, the MFEF-DCNet model also achieved excellent results in the later stages of training with the accuracy around 0.9551. The loss value curves for different models were shown in **Figure 6**. The loss values shown in the vertical coordinates in **Figure 6** indicated the difference between the model recognized outputs and the actual labels. A smaller loss value means a better performance of the model, while a larger loss value means a larger difference between the model recognized results and the real results. As can be seen from the training loss curve, MFEF-DCNet decreased the fastest in the first 5 epochs, and the loss value reached its minimum in the final stage of model training. After 100 Epochs of training, MFEF-DCNet had an accuracy of 0.9551 on the training set.

**Figure 7:**
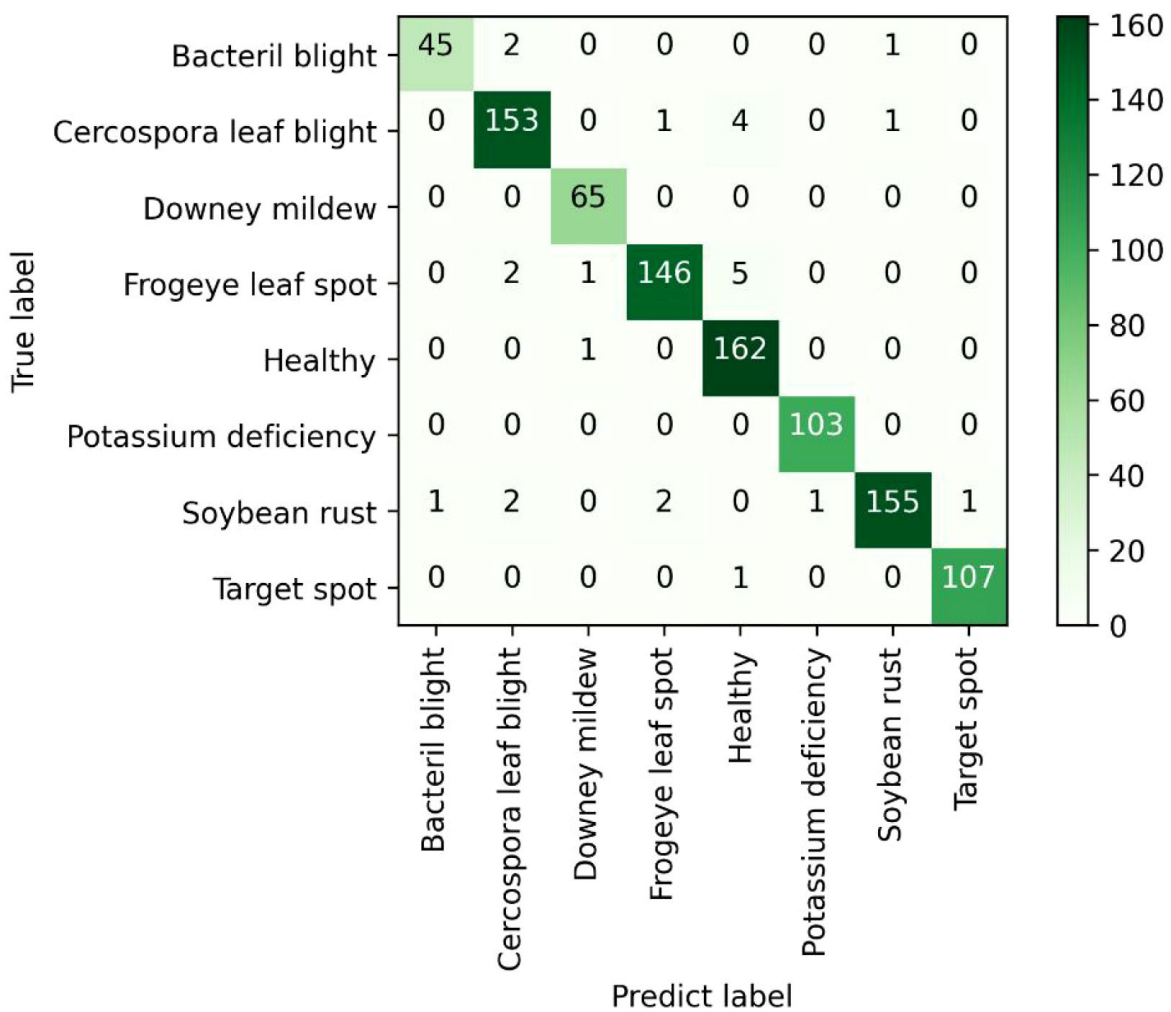
Confusion matrix.

After 100 epochs of training from scratch, the best trained weights were selected for all models and these weight coefficients were tested on a total of 962 test images that the models had never trained before. The test results of different models for different soybean leaf disease categories were shown in **Table 4**. It was evident from the table that the proposed MFEF-DCNet performs better than other models in the vast majority of recognition results. And the accuracy, macro accuracy, and weighted accuracy of the MFEF-DCNet model all greater than 0.94. In terms of the recognition results for the different soybean leaf disease categories, the MFEF-DCNet model had relatively poor recognition precision for the health category. In terms of recall, the MFEF-DCNet model had relatively poor recognition accuracy for frogeye leaf spot, and the model was able to correctly recognize 139 out of 154 disease images with a recognition recall of 0.9026. While the MFEF-DCNet model performed the best for downey mildew and potassium deficiency. All images of the two categories for soybean leaf diseases were correctly recognized with a recognition precision of 1.0000. In addition, the MFEF-DCNet model showed a small difference in recognition results between the different disease categories of soybean leaves. This indicated that the model achieved good performance for each category without overfitting or underfitting. Among the 962 test images, the MFEF-DCNet model correctly recognized a total of 911 images eight categories with an accuracy of 0.947. This result was the best among the all models used in this study. **Figure 7** showed the confusion matrix drawn by the MFEF-DCNet model after testing on 962 images. The numbers on the diagonal of the confusion matrix represented the counts that the recognitions of the model matched the true labels. From the confusion matrix, it was evident that the proposed MFEF-DCNet model achieved satisfactory results in all categories of test images.

**Table 4:** Classification training results of different network models.

	MFEF-DCNet			EfficientB0			ResNet50			VGG16			Xception			MobileNetV3Small			DenseNet201		
	P	R	F	P	R	F	P	R	F	P	R	F	P	R	F	P	R	F	P	R	F
Bacterial blight	0.8824	0.9375	0.9091	0.9388	0.9583	0.9485	0.7667	0.9583	0.8519	0.7581	0.9792	0.8545	0.8537	0.7292	0.7865	0.8250	0.6875	0.7500	0.8571	0.8750	0.8660
Cercospora leaf blight	0.9542	0.9182	0.9359	0.9172	0.9057	0.9114	0.8802	0.9245	0.9018	0.9221	0.8931	0.9073	0.9706	0.8302	0.8949	0.9286	0.8994	0.9137	0.8000	0.7547	0.7767
Downey mildew	1.0000	0.9385	0.9683	0.8025	1.0000	0.8904	0.9483	0.8462	0.8943	0.9365	0.9077	0.9219	0.9403	0.9692	0.9545	0.8871	0.8462	0.8661	0.8000	0.9231	0.8571
Frogeye leaf spot	0.9720	0.9026	0.9360	0.9433	0.8636	0.9017	0.9272	0.9091	0.9180	0.9085	0.8377	0.8716	0.7023	0.9805	0.8184	0.8411	0.8247	0.8328	0.6027	0.8766	0.7143
Healthy	0.8710	0.9939	0.9284	0.8333	0.9509	0.8883	0.9150	0.8589	0.8861	0.8466	0.9141	0.8791	0.9325	0.9325	0.9325	0.8041	0.9571	0.8739	0.8790	0.8466	0.8625
Potassium deficiency	1.0000	0.9903	0.9951	1.0000	1.0000	1.0000	0.9902	0.9806	0.9854	0.8879	1.0000	0.9406	0.9808	0.9903	0.9855	0.9619	0.9806	0.9712	1.0000	0.9806	0.9902
Soybean rust	0.9437	0.9321	0.9379	0.9675	0.9198	0.9430	0.9281	0.8765	0.9016	0.9632	0.8086	0.8792	0.9926	0.8272	0.9024	0.9130	0.9074	0.9102	0.9474	0.7778	0.8542
Target spot	0.9906	0.9722	0.9813	1.0000	0.8426	0.9146	0.8814	0.9630	0.9204	0.9558	1.0000	0.9774	0.9901	0.9259	0.9569	0.9474	0.8333	0.8867	0.9863	0.6667	0.7956
Accuracy	0.9470			0.9210			0.9096			0.9023			0.9033			0.8857			0.8254		
Macro Average	0.9517	0.9482	0.9490	0.9253	0.9301	0.9247	0.9046	0.9146	0.9074	0.8973	0.9175	0.9040	0.9204	0.8981	0.9040	0.8885	0.8700	0.8800	0.8591	0.8376	0.8396
Weighted Average	0.9497	0.9470	0.9473	0.9271	0.9210	0.9215	0.9125	0.9096	0.9097	0.9070	0.9023	0.9019	0.9203	0.9033	0.9056	0.8886	0.8900	0.8900	0.8518	0.8254	0.8292

P represented the precision, R represented the recall, and F represented the F1-score.

### 3.2 Ablation study and local data testing

The ablation experiments were conducted as shown in **Table 5**. As can be seen from the table, the standard convolutional model had 6.66 million parameters, with an accuracy rate of 91.79% and an F1-score of 91.97%. Using the standard convolutional model as a baseline, when the CBAM module was introduced separately, the number of model parameters increased by 0.09M, yet the accuracy and F1-score improved by 0.52% and 0.55%, respectively. When replacing the standard convolution module with a separable convolution module, the number of model parameters was significantly reduced by 83.4%, with the parameters of 1.11 million. Meanwhile, model accuracy and F1-score improved by 2.08% and 1.92%, respectively. When the separable convolution and CBAM modules were introduced simultaneously, the model parameters reduced by 82.0%, while the model performance was the best, with an accuracy and F1-score of 94.70% and 94.73%, respectively. The accuracy and F1-score were improved by 2.91% and 2.76%, respectively. The above results indicated that separable convolutions had a good effect on model parameter compression, while the CBAM module can further enhance the model’s recognition capabilities through feature enhancement. Therefore, the MFEF module constructed by combining separable convolution and CBAM could significantly improve the recognition performance of the model, which provided technical support for the deployment of lightweight disease recognition models in practical scenarios.

**Table 5:** Comparison of ablation study results.

Convolution	Separable convolution	CBAM	Total Parameters	Trainable params	Non-trainable params	Accuracy	Average Precision	Average Recall	Average Fl	Weighted Precision	Weighted Recall	Weighted Fl
✓			6,662,376	6,652,456	9,920	0.9179	0.9357	0.9322	0.9292	0.9334	0.9179	0.9197
✓		✓	6,750,568	6,740,648	9,920	0.9231	0.9477	0.9367	0.9383	0.9365	0.9231	0.9247
	✓		1,109,096	1,099,176	9,920	0.9387	0.9484	0.9346	0.9404	0.9405	0.9387	0.9388
	✓	✓	1,197,288	1,187,368	9,920	0.9470	0.9517	0.9482	0.9490	0.9497	0.9470	0.9473

Each row represents a set of experimental data, and a check mark in the table indicates that the corresponding module is enabled.

In order to further verify the adaptability and actual application effect of the MFEF-DCNet model, the 164 local images of soybean leaves, which were collected in Dayuzhuang, Lanqing Township, Zhengyang County, Zhumadian City, Henan Province, were input into the MFEF-DCNet model. The recognition results of the model were shown in **Table 6**. As shown in the table, the average accuracy of the MFEF-DCNet model for four categories of soybean leaves was 0.9024, and the macro-mean and weighted mean of the model were around 0.9. The results indicated that the MFEF-DCNet model had a satisfactory recognition effect for the local soybean leaves. At the same time, it was shown that MFEF-DCNet had better application effect in practical applications. It also showed that the MFEF-DCNet model had a favorable application effect in practical applications.

**Table 6:** Evaluation results of MFEF-DCNet model on different categories of local soybean images.

Disease category	Precision	Recall	F1-score	Support
Bacterial blight	0.9167	0.9167	0.9167	48
Frogeye leaf spot	0.9600	0.9231	0.9412	52
Healthy	0.9118	0.8857	0.8986	35
Soybean rust	0.7812	0.8621	0.8197	29
Accuracy	0.9024			164
Macro average	0.8924	0.8969	0.8940	164
Weighted average	0.9054	0.9024	0.9034	164

### 3.3 Visualization

**Figure 8:**
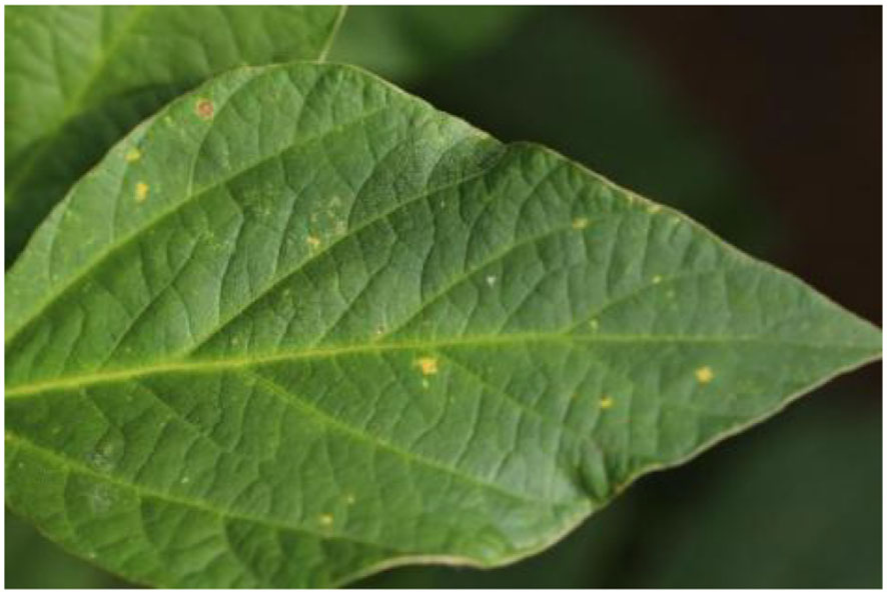
Soybean downy mildew leaves.

**Figure 9:**
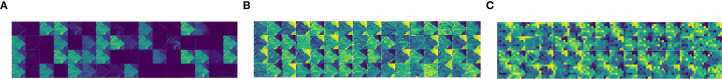
Characteristic map of soybean downy mildew in different periods: **(A)** Feature map (Early stage); **(B)** Feature map (Mid stage); **(C)** Feature map (Last stage).

Feature map visualization was a technique for understanding deep learning models, which is useful for observing the intermediate results produced by the model while processing the input data. By visualizing the feature map, the model’s ability to interpret the input data and extract features could be quantified and demonstrated, which in turn contributed to optimizing the performance of the model. **Figures 8** and **9** illustrate the process of visualizing the feature map of downy mildew using MFEF-DCNet. **Figure 8** showed the image of downy mildew as input to the neural network, and **Figure 9A**, **Figure 9B** and **Figure 9C** showed the feature maps of downy mildew at different depths of the model during the forward process [Early stage (**Figure 9A**), Mid stage(**Figure 9B**), Last stage (**Figure 9C**)]. According to the feature maps of different periods, it can be seen that the shallow information extracted by the model is mostly edges and textures. With the depth of the network, what MFEF-DCNet learns became more and more abstract, and the extracted features became richer and richer. However, the visual interpretability of these features was also getting decreasing.

**Figure 10:**
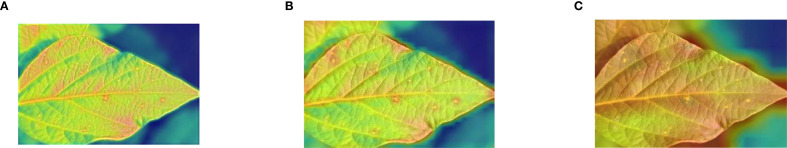
Heat map of soybean downy mildew in different periods: **(A)** Heat map (Early stage); **(B)** Heat map (Mid stage); **(C)** Heat map (Last stage).

By mapping the visualized feature map to the original image, the change of the network focus during the network training process could be observed. **Figures 10A**–**C** were the heat maps of different stages of the model by mapping the feature maps after visualization to the original image [Early stage (**Figure 10A**), Mid stage (**Figure 10B**), Last stage (**Figure 10C**)]. From the figures, we can see that the attention of the network gradually approaches towards the direction of the disease features during the learning process of the neural network.

According to the heat maps of different periods, it can be seen that the focus of the model was gradually approaching towards the direction of downy mildew disease characteristics in the process of neural network learning.

## 4 Discussion

The goal of this study was to develop an efficient, lightweight, and highly accurate network model for soybean leaf diseases identification. Based on the open-source dataset, operations such as rotating, cropping, and flipping the original images of soybean diseases were implemented by data balancing algorithms and image enhancement technology. These operations improved the balance of sample data while expanding the sample size. After that, the study constructed a multiscale feature extraction module for soybean leaves using CBAM and depthwise separable convolution, which was used to improve the feature extraction capability of the model. Meanwhile, the fusion among multi-scale feature layers was achieved by using dense connections in the model backbone network to improve the generalization ability. Existing soybean leaf pest and disease identification methods cannot simultaneously meet requirements in terms of identification accuracy (Kang et al., 2025) and model size (Miao et al., 2023). In comparison, several aspects such as model lightweighting, pest types, and accuracy were comprehensively considered in this study. After training and optimization, the MFEF-DCNet constructed in this study had the best results in terms of accuracy, model convergence speed, and precision, with the highest accuracy rate of 0.947, and is friendly for deployment on edge devices. The results of tests on multiple data sets indicate that MFEF-DCNet has good effectiveness and superiority in soybean leaf disease identification.

A lot of attempts were made during the construction and testing of the model. For example, several auxiliary classifiers were added to the model during the training process in order for the model to converge faster during the training process. However, the presence of the auxiliary classifiers had not enhanced the accuracy of the model from the training results obtained. Conversely, this operation consumed more arithmetic power. Therefore, no auxiliary classifiers were added to the final model construction. In addition, the residual structure was not used in the module when using the convolutional attention module. This is because the use of the residual structure was verified to have no significant improvement on the final model performance.

The MFEF-DCNet model proposed in this study achieved effective identification of eight diseases of soybean and achieved good results. However, the study in this paper still has some shortcomings and needs to be further improved in the future. The MFEF-DCNet model was able to classify each disease category in soybean leaf disease identification, but it was unable to realize the judgment of disease degree at the same time of disease identification. The essence of neural network classification is to assign pre-trained disease category probabilities to the image data of each input network. The disease category with the highest probability was ultimately selected as the disease category for the input image. Therefore, the model was only able to identify one of the diseases when multiple diseases are present in a leaf and is unable to accurately identify the other disease categories at the same time. Simultaneous identification of multiple diseases in a picture and the degree of disease was a direction for further research.

## 5 Conclusions

In this study, MFEF-DCNet, a lightweight deep convolutional neural network, was constructed for soybean leaf disease identification based on the open-source soybean leaf disease dataset. The training inference efficiency and identification accuracy of the model were improved by employing the soybean leaf multi-scale feature extraction fusion module, namely the dense connectivity and CBAM module. In comparison with other common identification models, MFEF-DCNet achieved a maximum accuracy of 0.947 while being lightweight. And its recognition accuracy for local soybean leaf diseases was 0.9024. Meanwhile, the heat map analysis showed that the leaf disease region features were correctly learned by MFEF-DCNet. Overall, future research would continue to deepen the problem of soybean disease recognition. More advanced deep learning techniques and methods would be attempted to contribute to the development and security of the soybean industry.

## Data Availability

The original contributions presented in the study are included in the article/supplementary material. Further inquiries can be directed to the corresponding authors.
